# Spring migration patterns of red knots in the Southeast United States disentangled using automated telemetry

**DOI:** 10.1038/s41598-023-37517-y

**Published:** 2023-07-10

**Authors:** Adam D. Smith, Felicia J. Sanders, Kara L. Lefevre, Janet M. Thibault, Kevin S. Kalasz, Maina C. Handmaker, Fletcher M. Smith, Tim S. Keyes

**Affiliations:** 1 U.S Fish and Wildlife Service, National Wildlife Refuge System, Inventory and Monitoring Branch, Athens, GA 30605 USA; 2 South Carolina Department of Natural Resources, 220 Santee Gun Club Road, McClellanville, SC 29458 USA; 3 Florida Gulf Coast University, Fort Myers, FL 33965 USA; 4 South Carolina Department of Natural Resources, 217 Fort Johnson Road, Charleston, SC 29412 USA; 5 U.S. Fish and Wildlife Service, Florida Ecological Services Field Office, 28950 Watson Blvd, Big Pine Key, FL 33043 USA; 6 University of Massachusetts Amherst, 160 Holdsworth Way, Amherst, MA 01003 USA; 7 Georgia Department of Natural Resources, 1 Conservation Way, Brunswick, GA 31520 USA; 8 Present Address: American Bird Conservancy, The Plains, VA 20198 USA; 9 Present Address: Thompson Rivers University, Kamloops, BC V2C 0C8 Canada

**Keywords:** Ecology, Zoology

## Abstract

Red Knots use the Southeast United States as a stopover during north and southbound migration and during the winter. We examined northbound red knot migration routes and timing using an automated telemetry network. Our primary goal was to evaluate the relative use of an Atlantic migratory route through Delaware Bay versus an inland route through the Great Lakes *en route* to Arctic breeding grounds and to identify areas of apparent stopovers. Secondarily, we explored the association of red knot routes and ground speeds with prevailing atmospheric conditions. Most Red Knots migrating north from the Southeast United States skipped or likely skipped Delaware Bay (73%) while 27% of the knots stopped in Delaware Bay for at least 1 day. A few knots used an Atlantic Coast strategy that did not include Delaware Bay, relying instead on the areas around Chesapeake Bay or New York Bay for stopovers. Nearly 80% of migratory trajectories were associated with tailwinds at departure. Most knots tracked in our study traveled north through the eastern Great Lake Basin, without stopping, thus making the Southeast United States the last terminal stopover for some knots before reaching boreal or Arctic stopover sites.

## Introduction

Effective management of migratory species depends on a nuanced understanding of their movement pathways through space and time. Arctic nesting shorebirds make some of the longest journeys of any species on earth, flying extreme distances in the spring from wintering sites to nesting sites with limited, predictable stopover sites *en route*^1^. In response to the many factors that influence shorebird stopover locations and migration routes, such as flight speeds, refueling rates, optimal arrival at breeding territories, and mortality risks^2,3^, shorebirds have evolved flexibility in their migration strategies, with demonstrated variation even within the same population^4^.

The *rufa* subspecies of the red knot (*Calidris canutus rufa*), hereafter “red knot(s)” or “knot(s),” breeds in the Canadian Arctic and winters in four distinct areas: the Southeast United States/Caribbean, the north coast of South America, the western Gulf of Mexico/Central America, and in southern South America^5,6^. The Southeast United States/Caribbean hosts one quarter of the total estimated wintering knot population^5^. Red knot winter abundance at the southern tip of South America declined by 75 percent from 1980 and 1990s to 2018–2020^5^. Spring 2021 surveys of Delaware Bay, New Jersey USA, a crucial staging site for northward migrating red knots, produced the lowest red knot numbers since 1981^7^. Consistent declines in red knot population estimates resulted in the species being listed as threatened in the United States in 2014 and endangered in Canada in 2020^8,9^.

Delaware Bay is the final stop for many thousands of red knots before a single direct flight to boreal and Arctic staging grounds, yet research in the last decade has identified additional stopover sites and illustrated diverse migration routes for knots using the Western Atlantic flyway^10,11^. For example, the Southeast United States (North and South Carolina, Georgia, and Florida) has been identified as a critical region for knots as a stopover during north and southbound migration and during the winter^12–14^. The most recent U.S. Fish and Wildlife Service estimate for the red knot population is 63,600^5^. The southbound passage population utilizing the Georgia coast was estimated to be approximately 23,400 knots^12,15^, while the Kiawah Island, Seabrook Island and Deveaux Bank area of South Carolina, supports a minimum spring passage population of more than 17,000 individuals^13^. This suggests the Southeast United States supports upwards of a third of the knot population during migration each year.

Within the Southeast United States, the spatial distribution of knots in the winter can shift interannually, mainly between Florida and Georgia^6^. Red knots using the Southeast United States during winter and spring use at least two different northbound migration routes. Knots leave the Southeast United States and then stop along the mid-Atlantic (e.g., Virginia or Delaware Bay) before departure overland towards boreal and Arctic stopover sites, or travel via an inland route with overland departure directly from the coast of the Southeast United States towards central Canada and then the Arctic^10,16,17^. Resighting of knots who spend the winter in southwest Florida suggest that they regularly use the coastal route via Delaware Bay^14^, although this could be due to disproportionate resighting effort in Delaware Bay rather than indicative of a migratory route preference. For example, all three knots providing geolocator tracking data, took an inland migration route from South Carolina to their next stopover in boreal or Arctic habitat^13^.

We examined northbound red knot migration routes and timing from the Southeast United States using the Motus Wildlife Tracking System, hereafter Motus^18,19^, an automated telemetry network that enables continental scale tracking studies of species when other tracking technologies are not available due to size or cost limitations. Our primary goal was to evaluate the relative use of an Atlantic migratory route through Delaware Bay versus an inland route through the Great Lakes *en route* to Arctic breeding grounds and identify areas of apparent stopovers. Secondarily, because migration routes and departure decisions evolved in part to use beneficial atmospheric conditions e.g., wind assistance^20–22^, we explored the association of red knot routes and ground speeds with prevailing atmospheric conditions.

## Methods

### Study sites

Knots present in Florida in March mainly winter in the Southeast United States and Caribbean but not necessarily in Florida^10^ and knots stopping in South Carolina in April and May come from all four wintering populations^8,13,23^. We thus focused our work primarily on red knots using the South Carolina coast in April and May, and to a lesser extent the Gulf Coast of Florida. In April 2017 and 2018 we captured red knots roosting in flocks of up to 3,000 individuals on Seabrook Island, (32°34′N, 80°9′W) in Charleston County, South Carolina. Seabrook Island and adjacent Kiawah Island, during the last decade, have supported spring flocks of over 8000 red knots^24^. Both Kiawah and Seabrook Islands are recreational destinations with substantial beach traffic in April and May. During May 2019 we captured red knots feeding on horseshoe crab (*Limulus polyphemus*) eggs at Deveaux Bank Seabird Sanctuary (32°32′N, 80°11′W), a nearshore island owned by South Carolina Department of Natural Resources and primarily managed for waterbird conservation. We captured a single red knot in May 2019 at Turtle Island Wildlife Management Area in Jasper County, SC (32°4′ N, 80°53′ W). Florida captures were restricted to a single season in March 2019 at Fort de Soto Park in Pinellas County (27°37′N, 82°43′).

### Bird capture and tag deployment

Red Knots were captured in predominantly single species flocks with cannon nets, removed immediately, and placed in holding cages for processing. Birds were aged using plumage characteristics described in Pyle^25^. We recorded morphometric measurements, weighed, and fitted each knot with a USGS incoloy band and a uniquely inscribed 3-character flag. To prepare a bird for transmitter attachment, we clipped a small patch of feathers above the uropygial gland, and affixed the digitally coded VHF transmitters (166.38 MHz with 4.7 to 11.3 s burst intervals; NTQB2-4-2, Lotek Wireless; hereafter “nanotag”) with a polyacrylamide glue^26^. Nanotags weighed approximately 1 g (< 1% of red knot body mass) and measured 12 × 8 × 8 mm with an 18 cm long external wire antenna.

### Processing detection data

We processed data following methods of prior studies using Motus^21,27,28^. Tag identity is encoded in the duration of three rapid, consecutive pulses comprising a single tag ‘burst’ in combination with the precisely fixed interval between these bursts; the pattern of pulse lengths and burst interval is unique among tags. We eliminated false detections during post-processing by examining all detections with less than three consecutive pulses and considering several derived metrics of detection structure related to a tag’s frequency, burst interval, and other signal qualities, as well as considering the noise context of the receiving station and other valid detections of the tag.

### Departure dates

We examined the detection history of each individual red knot to calculate the day of departure from the South Carolina coast (but not for the smaller sample of Florida birds). To estimate the departure window from coastal South Carolina to northern destinations we used two methods. First, for birds that were detected by a station away from the coast within South Carolina or North Carolina (n = 10), we considered the date of first detection away from the capture site as the initiation of migration. At the time of this study only one (2017), two (2018) and six (2019) receiving stations were operational in South Carolina, and none within 75 km of the capture location. The lack of stations near the capture location made it difficult to estimate departure date precisely for most individuals. To make use of all birds detected during northbound migration, we thus assigned the 37 knots with uncertain departure dates a range of potential departure dates. We assigned each day between the tag deployment date and the date of first detection away from the capture site a percentage that reflected the possibility the bird departed on that day. For example, a knot with a known departure date made its full contribution (1) to a date, whereas a knot with a 5-day potential departure window contributed 0.2 to each of the days in that date range. Summing these departure date weights for all 47 individuals produced an estimated departure timeline for our full data set while accounting for uncertainty in exact departure dates.

### Migration pathways

Similar to Sanders et al.^29^, we compared the relative use of the Atlantic Coastal route through Delaware Bay versus an inland route using patterns of tag detections in two primary watersheds: Delaware Bay, which we defined as any stations within 30 km of the (HUC 02040204) Delaware Bay Hydrological Unit^30^, and the Great Lakes Basin as the 5 lakes and their associated subbasin watersheds^31^. Migratory routes were typically quite distinct in the detection dataset and were scored as follows. We classified individuals with multiple detections within Delaware Bay separated by more than one day to have “stopped” in Delaware Bay. We classified a single individual to have “likely stopped” in Delaware Bay as it was detected multiple times over two or more days but just outside our defined watershed. An individual was deemed to have “skipped” Delaware Bay when the detection history indicated a direct, or nearly so, flight between the coast of South Carolina and a station north of Delaware Bay without time for a stopover in Delaware Bay. An individual was scored to have “likely skipped” Delaware Bay if they were not detected within, or only during a direct flight through, the Delaware Bay watershed and the detection history suggested time was too constrained for a stop in Delaware Bay, or the trajectory of the detection path seemed inconsistent with use of the mid-Atlantic coast. We classified use of Delaware Bay as “unknown” when a red knot was never detected in Delaware Bay but the spatiotemporal patterns of detection lacked sufficient resolution to distinguish between strategies. We explored a possible relationship between individual body condition and migratory strategy with a linear model of body mass at capture and assigned migration strategy.

### Stopovers

We defined stopover locations, based on detections more than 50 km from the tagging location, as either detections at a single station spanning > 4 h with no intervening detections elsewhere, or spanning > 6 h among multiple stations within 30 km of each other^27^. Although Crysler et al.^27^ identified stopovers as detections at a single station spanning > 8 h (with no intervening detections elsewhere) or spanning > 10 h among multiple adjacent stations, we relaxed those timeframes because shorebirds migrating during the day may elect to temporarily rest or refuel (i.e., mix the acts of migration and stopover^32^) for shorter time periods. Bayly et al.^33^ proposed distinguishing between “true” stopover during multiday refueling stops and brief stops to rest stops of < 24 h, but that finer-scale distinction is beyond the scope of this dataset. To visualize areas that were used repeatedly for stopovers, we buffered the stations associated with stopover detections for each individual by 50 km and overlaid the resulting polygons to generate a rough stopover “heat map.”

### Speed of travel and wind assistance

We estimated migration speeds for knots during migratory flights using detection times at receiving stations separated by at least 150 km (to exclude local movements and reduce bias from uncertainty in an individual’s precise location during a detection^28^), and restricted estimates of migration speed to flights of 18 h or less. This resulted in 40 flight trajectories representing 30 individuals for analysis of travel speed and wind assistance.

We calculated total trajectory length as the shortest distance connecting all receiving stations passed during the flight between the beginning and ending receiving stations, and trajectory displacement length as the shortest distance between the beginning and ending receiving station, using the ‘trajr’ package^34^ in R (version 4.1.2^35^). We calculated net ground speed for a flight as the trajectory displacement length divided by the time between the last detection at the beginning receiving station and the first detection at the ending receiving station. We permitted an individual to contribute multiple flights, provided there was no receiver station overlap in the trajectories. For each migratory flight, we also estimated tailwind support at departure using surface wind conditions (i.e., 1000 hPa pressure level) at the measurement time closest to departure. We assumed the “preferred” direction of movement (as required by the calculation of tailwind support) was the bearing that would take the bird to the ending receiving station following a great-circle route. Tailwind was measured as the velocity of supporting wind movement in that “preferred” direction, and any wind supporting the flight to within 90 degrees of the direction of movement was considered a tailwind (e.g., winds from just south of east through to just south of west would technically be tailwinds for a due north flight). We acquired wind component data for each migratory flight from the NCEP/NCAR Reanalysis project^36^ via the RNCEP package^37^ in R (version 4.1.2^35^.

### Ethical approval and consent to participate

Nanotag attachment in South Carolina was approved by the U.S. Geological Survey, bird banding laboratory, federal bird banding permit number: 06658, issued to the South Carolina Department of Natural Resources. Nanotag attachment in Florida was approved by the U.S. Geological Survey, bird banding laboratory, federal bird banding permit number: 24188, issued to Kevin Kalasz. All methods were performed in accordance with the relevant guidelines and regulations of the Bird Banding Laboratory.

## Results

We deployed nanotags on 108 red knots. In coastal South Carolina, we tagged 96 red knots in 2017 through 2019 (spanning 19 April–24 May; median deployment date of 6 May across all 3 years): 72 on Deveaux Bank, 23 on Seabrook Island, and one at Turtle Island. We deployed transmitters on 12 red knots at Fort De Soto, FL in March 2019. Most red knots were aged as “after second year” (adult) based on plumage characteristics (n = 93), 11 as second year (SY) (i.e. hatched in previous year), and unknown in 4 individuals. 73 red knots (68%) were detected by at least one receiving station in the Motus network, although only 51 (47%; 48 adult, 3 SY) were detected north of South Carolina during northbound migration (defined as deployment to 15 June) and provided information for one or more analyses. We constrain our discussion and conclusions in this study predominantly to the 48 adult birds. Average body mass at transmitter deployment for 96 knots tagged in South Carolina was 137 ± 15 g (median = 138 g).

### Departure dates

**Figure 1:**
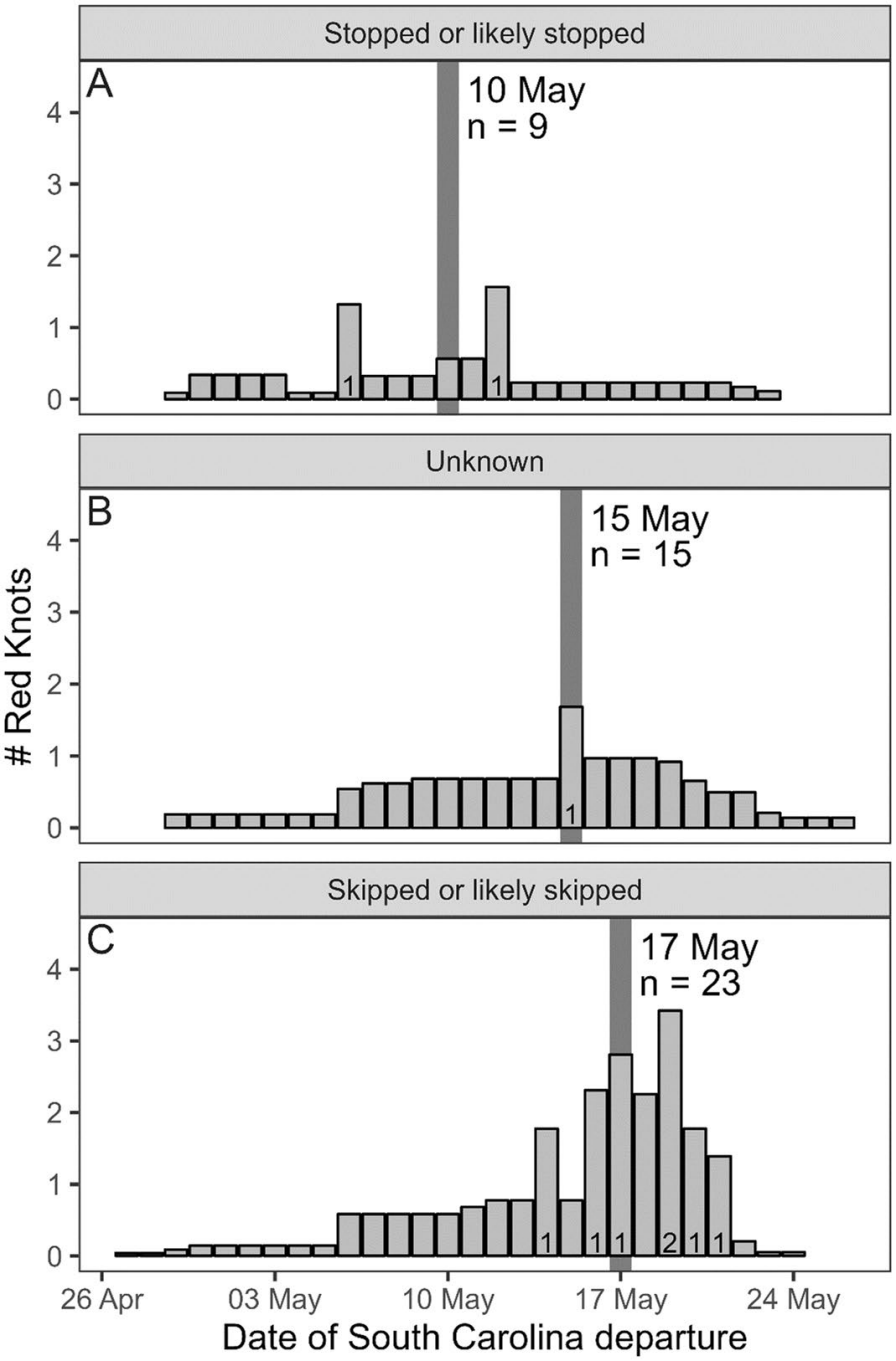
The departure date for 47 adult red knots migrating northward from coastal South Carolina that (**A**) stopped or likely stopped in Delaware Bay, (**B**) used an unknown migratory route, or (**C**) skipped or likely skipped Delaware Bay. The number of red knots with known departures on specific dates is indicated at the bottom of a given bar (n = 10 across all strategies). The weighted median date of departure is indicated by the gray vertical bar. This figure was created in R (version 4.1.2), available from https://www.r-project.org.

Precise departures dates from South Carolina could be calculated for only 10 adult red knots. For the 37 other adult knots detected during northbound migration, we incorporated uncertainty using ranges of possible departure dates that spanned 2–27 days. The weighted median departure date for birds stopping in Delaware Bay was earlier (May 10, Fig. 1A) than birds skipping Delaware Bay (May 17; Fig. 1C), although there was considerable overlap. Departure dates of red knots using an unknown migratory route overlapped other departures as well, with a median date of departure of May 5 (Fig. 1B). A single red knot departed overland from Florida through the Great Lakes and did not contribute a departure date for this analysis.

### Migration pathways

**Figure 2:**
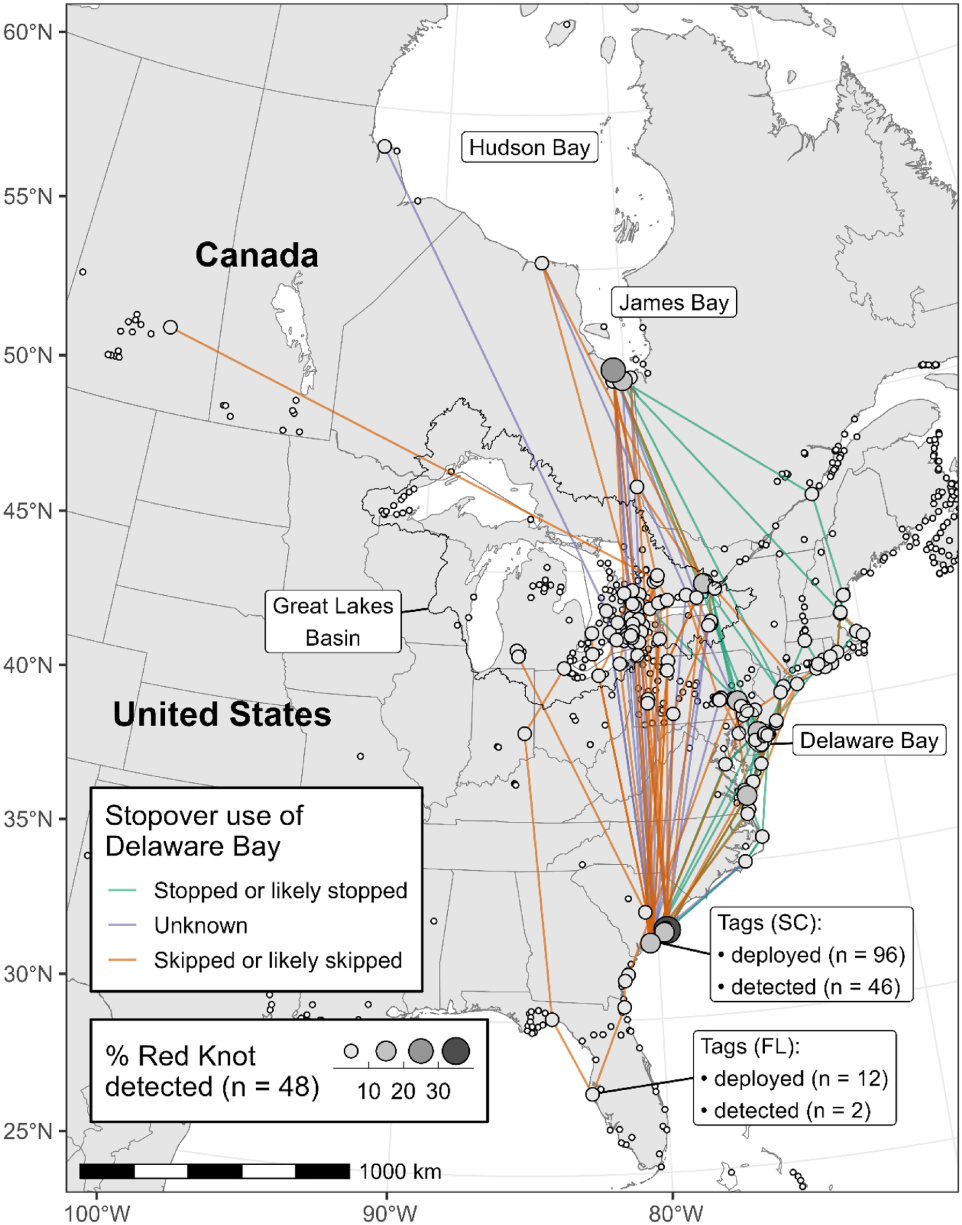
Locations of active Motus receiver stations (white dots with black outlines), and the northern migratory trajectories of 48 adult Red Knots tagged in South Carolina during April and May (2017–2019) and Florida in March 2019 and detected during northbound migration. Estimated stopover used of Delaware Bay is described in the text. The lines connect detections of individuals between receiving stations or between the tagging location and a receiver station. These tracks represent simplified flight trajectories and not necessarily actual fight paths. Circle diameters reflect the number of individual birds detected by a given receiving station. This map was created in R (version 4.1.2), available from https://www.r-project.org.

Among the 48 adult red knots detected in the Motus network that provided detection information north of South Carolina, 33 individuals provided detection information sufficient to evaluate whether there was passage and stopover in Delaware Bay. Most skipped or likely skipped Delaware Bay (73%, 24 of 33 birds) while the balance stopped or likely stopped in Delaware Bay (27%, 9 of 33 birds) for at least 1 day (Fig. 2). We were unable to assign nine knots confidently to either migration strategy. Nearly half of adult red knots were detected in either James Bay (38%, 18 of 48 birds) or Hudson Bay (8%, 4 of 48 birds); no knots were detected in both locations (Fig. 2). The first day of detection in either Hudson Bay or James Bay ranged from 19 May to 7 Jun (median 24 May).

Most birds that skipped Delaware Bay traveled north, or appeared to, from the South Carolina coast through the eastern Great Lake Basin, although a few individuals used an Atlantic Coast strategy that did not include Delaware Bay, relying instead on the areas around Chesapeake Bay or New York Bay for stopover (Fig. 2). Several knots that stopped in Delaware Bay also stopped in and used those other Atlantic areas (versus Delaware Bay exclusively), including a few birds that used the New England Coast after leaving Delaware Bay. We identified three birds that headed south to Georgia following tagging in South Carolina, but we did not get additional information about these birds’ spring movements.

**Figure 3:**
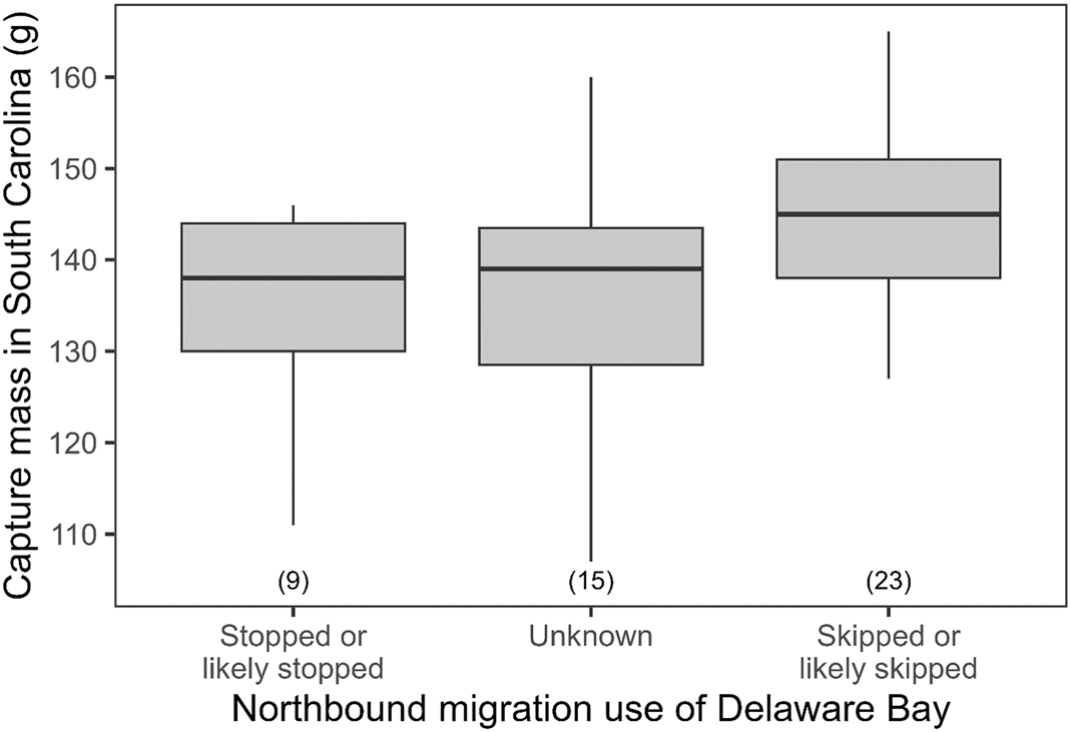
Comparison of body mass (g) at capture for 47 adult Red Knots categorized by their inferred use of Delaware Bay during northbound migration after departing from coastal South Carolina. The number of individuals in each category is indicated in parentheses. This figure was created in R (version 4.1.2), available from https://www.r-project.org.

Red knots inferred to have skipped Delaware Bay were approximately 9 g heavier on average at the time of capture compared to those inferred to have stopped there and those with an unknown migration strategy (F_2, 44_ = 3.39, *P* = 0.04). However, there was considerable overlap in body mass among the three groups and migration strategy explained only about 10% of the variation in capture mass (Fig. 3).

### Stopover

**Figure 4:**
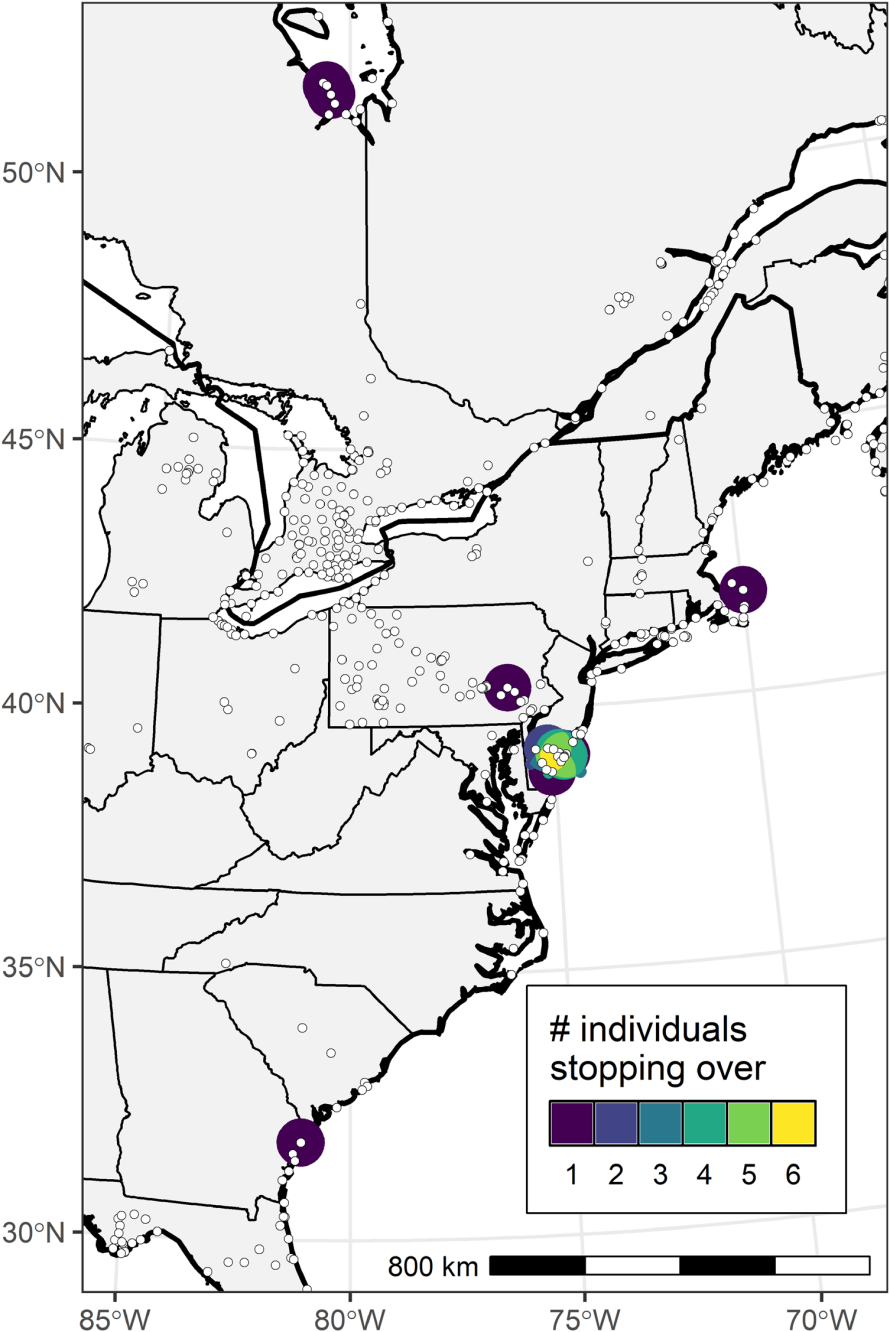
Locations of active Motus receiver stations (white dots with black outlines) and stopover locations for 11 adult Red Knots. Color indicates the number of individuals that used a location for a stopover. This map was created in R (version 4.1.2), available from https://www.r-project.org.

We identified stopover events for 11 adult red knots. These were largely concentrated in Delaware Bay (n = 6), with single stopovers identified in coastal Georgia, eastern Pennsylvania, Cape Cod, Hudson Bay in eastern Ontario (Fig. 4), and eastern Saskatchewan (not illustrated). The stopover in Georgia represented a bird tagged in SC that moved south along the coast at least 100 km before resuming northbound migration. Notably, we did not detect any stopovers in the Great Lakes Basin. In the Great Lakes Basin, all detection windows were less than 1 day (< 6 h, in fact; n = 30), suggesting that adult knots move quickly through that region. Note that this pertains only to adult red knots departing from the southeast Atlantic coast during the last two weeks of May.

In some cases, the short duration of red knot presence in the Great Lakes was corroborated by subsequent detections of the same individuals farther north in either James Bay or Hudson Bay (n = 9). For example, five of these individuals were detected in James Bay or Hudson Bay < 30 h after their last Great Lakes detection, providing confirmation of the limited stopover use of the Great Lakes Basin inferred from detection histories. However, the remaining four knots were detected in James/Hudson Bay 4–17 days later, suggesting that they went undetected for some time north of the Great Lakes Basin or stopped somewhere between the Great Lakes Basin and James/Hudson Bay.

**Figure 5:**
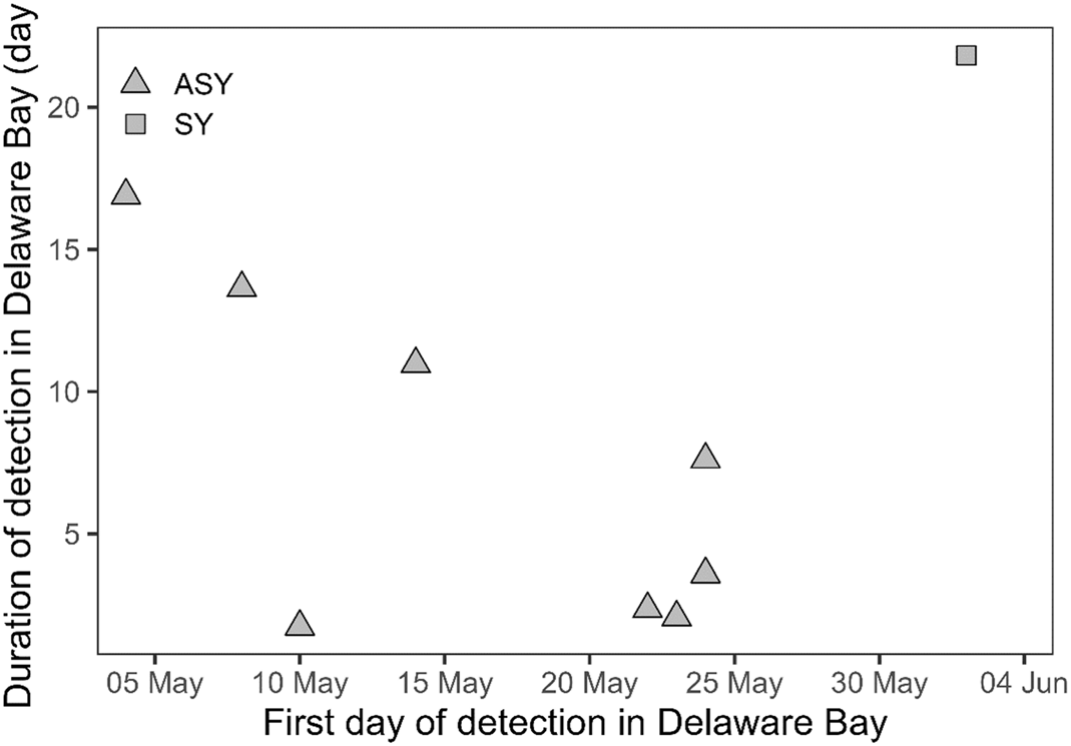
Stopover duration (time elapsed between first and last detection) relative to arrival date in Delaware Bay for 8 adult and 1 s year red knots. This figure was created in R (version 4.1.2), available from https://www.r-project.org.

Stopover duration in Delaware Bay (measured as time elapsed between first and last detection) for adult red knot averaged 7.4 ± 5.9 days (median: 5.6, range = 1.7–16.9 d, n = 8), which varied with date of first detection in the Bay. Adult knots detected at Delaware Bay earlier in spring tended to stay longer (linear model: t_6_ = − 2.4, *P* = 0.05; R^2^ = 0.4; Fig. 5). One knot arrived in Delaware Bay on 10 May and stayed less than 2 days; this individual moved north from Delaware Bay to spend nearly two weeks in lower New York Bay. One second year knot arrived in Delaware Bay in early June and was detected in this region until June 23, a clear contrast to the patterns of adult detection in Delaware Bay (Fig. 5).

### Migration speeds and wind assistance

**Figure 6:**
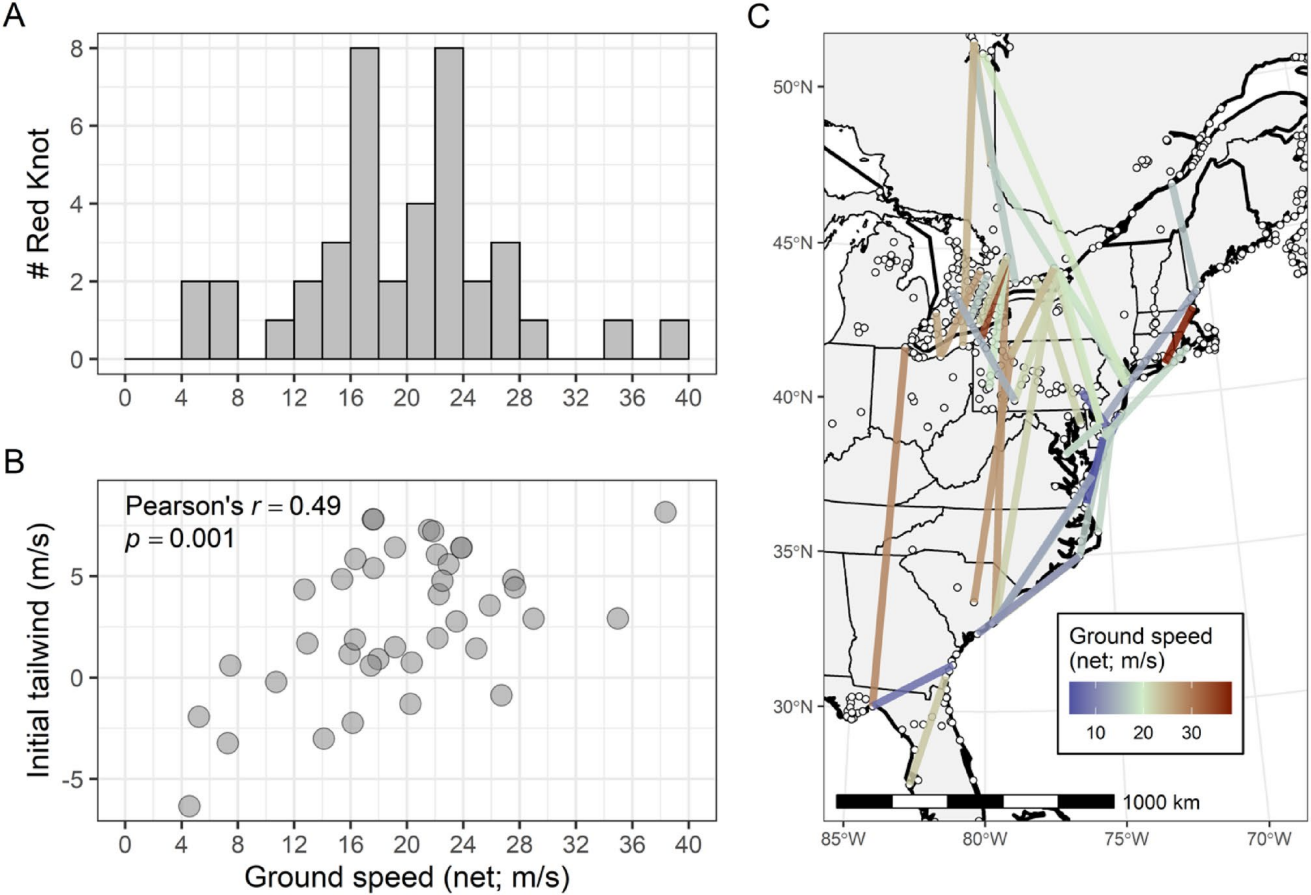
(**A**) Estimated ground speeds of red knot flights (n = 40 flights of 30 individuals) tagged during Spring migration on the Atlantic Coast of South Carolina (April to May, 2017–2019) and the Gulf Coast of Florida (March 2019). (**B**) Relationship between estimated ground speed of red knot flights and surface tailwind at the initiation of the flight. (**C**) Map depicting estimated ground speeds of red knot flights; white points represent Motus receiving stations active at any time during the study. This figure was created in R (version 4.1.2), available from https://www.r-project.org.

The median total trajectory length of measured migratory flights was 485 km (range: 161–1342 km); median trajectory displacement (i.e., the orthodrome distance) between beginning and ending receiver stations was 450 km (range: 158–1299 km). Estimated net ground speed along 40 flight trajectories representing 30 individuals averaged 20 m/s (Fig. 6a). Ground speeds were positively correlated with tailwind support towards the ending station (Pearson’s r = 0.49, *P* = 0.001; Fig. 6b). Nearly 80% of migratory trajectories were associated with tailwinds at departure. There seemed to be some general geographic patterns in red knot ground speeds, with lower-than-average speeds associated more with flights along the Atlantic Coast (South Carolina to Maine), and higher speeds associated more with overland flights from the coast towards and through the Great Lakes Basin (Fig. 6c). Flight from the mid-Atlantic Coast towards boreal and Arctic stopover sites tended to be representative of more average ground speeds.

## Discussion

Motus tracking in this study showed that red knots using South Carolina in the spring took a variety of northern routes but primarily migrated over land through the Great Lakes towards breeding grounds rather than along the Atlantic coast. Additionally, one of three knots tagged in Florida that provided migration route information flew directly to the southern Great Lakes, its final detection, from its tag deployment location in Florida. Most knots tracked in our study did not stop in the Great Lakes, thus making South Carolina (and the Southeast United States) the last terminal stopover for some knots before reaching boreal and Arctic habitats. Faster flight speeds toward and through the Great Lakes compared to lower speeds along the Atlantic coast support our inference that individuals utilizing this strategy made no or few stops during an inland route toboreal and Arctic stopover sites.

A smaller portion of knots in our study were detected at Motus stations on the Atlantic coast of the U.S. north of South Carolina, including approximately a quarter of the knots likely stopping in Delaware Bay. This study demonstrates the diversity of red knot spring migration routes with some knots leaving directly from the Southeast to boreal and Arctic stopover sites and others using a wide array of known stopover sites along the Atlantic U.S. coast before flying to breeding grounds. Similarly, banded wintering knots in Florida were subsequently resighted from Georgia to Massachusetts^14^. Multiyear migration tracks of individual knots with geolocators show migration routes and stopover sites may vary interannually^10,13^. For example, one knot tracked with a geolocator for multiple years flew directly to boreal and Arctic stopover sites from South Carolina and the following year left South Carolina and stayed three days in North Carolina before flying north^13^. Other individuals may stop in Delaware Bay one year but not the following year^10^. Many factors such as food availability, predation risks, competition, habitat preferences, breeding ranges, and genetics affect long-distance migration routes^38^ thus variation between individuals and even within individuals from year to year is not surprising. Although individuals demonstrate variation in migratory routes, a recent analysis of resighting data found that knots in the Southeast United States are 75% site faithful to specific sites (within a 30 km radius) from one year to the next, yet, similar to our study, they also found within-year movement between sites in South Carolina, Georgia, and Florida^39^. First-time comprehensive aerial surveys of South Carolina and Georgia in 2022 found knots widespread, not just concentrated in our study area, also emphasizing the area’s importance for knots (SCDNR and GADNR unpubl. data).

Knots caught in May in Delaware Bay were found to winter in Argentina, Chile and near Florida^40^ and knots stopping in Georgia migrating south represented all wintering populations^12^. The Southeast United States and Caribbean wintering population is thought to be stable, but the southern South America wintering population continues to decline^7^. To better understand the importance of the Southeast United States for knot spring migration, future research should include determining wintering origin for knots using this region.

### Mass

In our study, knots that skipped Delaware Bay were slightly heavier at capture time than those that stopped in Delaware Bay or had an unknown migration strategy. The ability to gain sufficient weight during northward migration is crucial, and a shortage of food resources in Delaware Bay has been linked to reduced subsequent adult survival and reproductive success of red knots^41^. A larger sample size overlaid with studies of food availability in the Southeast United States will help us better understand the role of weight on an individual’s decision to move to another stopover site to gain adequate departure mass versus completing refueling in the Southeast United States and departing directly from there to central Canada.

### Stopovers

The average stopover in Delaware Bay in this study (7 days) was shorter than the average of 11–12 days (range 2–22) reported in a larger Delaware Bay tagging project^42^. This difference may be a result of our small sample size (N = 9) or because knots in our study utilized Delaware Bay as a secondary stopover, after having stopped at least one other site along the U.S. Atlantic coast, rather than only stopping in Delaware Bay. As expected, knots that arrived earlier to Delaware Bay stayed longer than knots that arrived later, because Delaware Bay is the final stop for many knots before arriving in boreal andArctic habitat from late May to early June^6,17^. Immature knots do not migrate to nesting grounds but stay at more southern locations^6^, a likely explanation for the SY knot in our study that stayed in Delaware Bay at least until June 23.

Knots that departed the Southeast United States via an inland route not only bypassed Delaware Bay but made no or very brief stopovers in the Great Lakes Basin. Similarly, Ruddy Turnstones tagged in late May in South Carolina flew directly through the Great Lakes region toward boreal and Arctic habitat and none were detected on the Atlantic coast between South Carolina and New Jersey^29^.

Almost half the knots in this project were detected in James Bay or Hudson Bay. As in other tracking projects, our study showed the west coast of the Hudson Bay and James Bay are spring stopover sites for red knots using the both the Mid-Continent and West Atlantic Flyways^10,13,43,44^.

### Wind support and flight speed

Birds, including shorebirds, may make use of tailwinds to assist in migration, and departure timing may be influenced by timing of optimal tailwind conditions^20–22^. Knots making use of advantageous winds at departure can arrive at breeding grounds earlier. Red Knots in our study made use of tailwinds at departure from South Carolina, similar to findings that knots with higher mass captured in Delaware Bay had more tailwind support along their entire migratory path^21^. Further investigations on how weight and wind influence the migratory decisions of knots are needed to understand the diverse options knots face along their northbound migratory routes.

### Conservation

This project identified the Southeast United States as a critically important spring stopover site for red knots. Conservation of important beaches in this area must be a priority for halting further population declines. Knots only stop at sites with high quality prey to speed up their migration time^45^. Spring staging sites provide food that will fuel flights toward Arctic breeding grounds^46^, and for survival in cold high latitude conditions upon arrival^47^. Our study emphasizes the need to prioritize efforts that will maintain stable spring food resources in the Southeast United States. *Donax* clams and horseshoe crabs^48^ should be prioritized because knots utilize these prey resources to undertake non-stop migration from the Southeast to the breeding grounds.

Population estimates and trends for red knots using the Western Atlantic Flyway are determined by spring surveys of Delaware Bay and Virginia^16^. This study shows a portion of knots do not use either of these regions, highlighting the need to expand the geographic regions included in these estimates. The diversity of spring stopover sites used by red knots must be incorporated in survival and recruitment estimates as well as ongoing population monitoring.

This research demonstrates the usefulness of Motus for tracking the migration of red knots. While the percentage of tagged knots we detected (68%) is lower than the detection rate of knots tagged in the spring in Delaware Bay (84%), 42% of the Delaware Bay tagged knots were only detected once and mostly near the capture location within the dense network of stations around Delaware Bay^21,28^. For the 35 Red Knots not detected in this study, the most likely explanation for lack of detection is tag loss. We attached transmitters with glue only, which potentially left them susceptible to removal by repetitive preening or body molt. Other possible explanations for lack of detections include death due to natural causes and the use of a migratory route with low network coverage (e.g., through the western Great Lakes basin). Red knot tracking studies are important for identifying important sites for conservation and land use, such as determining knots’ exposure to offshore wind facilities and wind collision risks^49^. Strategic expansion of the Motus network in the Americas and beyond offers substantial potential to explore and understand the full life cycle of migratory shorebirds to support informed conservation decisions. Although much research is needed to understand the reasons knots use a variety of stopover sites^1^, future conservation planning must include the full network of sites that support the varied migratory routes and strategies used by this declining shorebird species.

## Data Availability

All code and data necessary to reproduce the analyses and figures are available via Figshare (10.6084/m9.figshare.23193842).
